# Points to Consider in the Development and Information Provision of Vaccines for Vaccination during Pregnancy: A Survey

**DOI:** 10.3390/vaccines10101684

**Published:** 2022-10-09

**Authors:** Yumiko Nomura, Yuusuke Oohashi, Mayumi Shikano

**Affiliations:** Graduate School of Pharmaceutical Sciences, Tokyo University of Science, Tokyo 162-8601, Japan

**Keywords:** vaccines for infectious diseases, vaccination during pregnancy, vaccine development, regulatory science

## Abstract

This report surveyed vaccination decisions during pregnancy based on the package inserts of vaccines approved in Japan, the USA, and Europe. Furthermore, it evaluates vaccination decision-making factors based on the characteristics of the target infections and the modality of the vaccines. Live vaccines known to cause fetal abnormalities are contraindicated for pregnant women, whereas vaccines for life-threatening infectious diseases are authorized for administration during pregnancy when the need is recognized, even for live vaccines. We compared the World Health Organization and European Medicines Agency guidelines on the development of vaccines for pregnant women and surveyed the details of the studies to collect information on SARS-CoV-2 vaccination during pregnancy. In compliance with the guidelines, for all SARS-CoV-2 vaccines, non-clinical reproductive and developmental toxicity studies and clinical trials including non-pregnant women of childbearing age were conducted prior to the vaccination of pregnant women. For all vaccines, information from registries on vaccination during pregnancy are used for post-marketing surveillance. While it is desirable to vaccinate women before pregnancy through planned immunization, whenever possible, pandemics such as H1N1 influenza and COVID-19 may require vaccination even during pregnancy. Necessary and sufficient studies for the decision of vaccination during pregnancy should be carried out promptly.

## 1. Introduction

Special precautions are taken while administering pharmaceutical drugs during pregnancy because of concerns regarding drug-induced fetal teratogenicity [1]. Moreover, vaccines for the prevention of infectious diseases are generally administered to healthy individuals, and the risks and benefits of vaccines may differ from those of therapeutic drugs [2]. Therefore, it is desirable to vaccinate women before pregnancy through a planned immunization program whenever possible.

It is important to formulate vaccination programs not only for children and older adults but also for women of childbearing age. However, considering diseases such as pertussis [3] and COVID-19 [4], vaccination during pregnancy is recommended, because the transferable maternal antibodies to newborns can protect them against infection until they are vaccinated. It is important to clarify the information affecting the decision to vaccinate safely and appropriately during pregnancy in order to prevent infectious diseases and activate immunity in newborns when necessary.

Cases have been reported on the vaccination experience of pregnant women for individual vaccines. In addition, review articles have revealed findings on vaccination of pregnant women for several vaccines that are expected to be administered to pregnant women, such as influenza, pertussis, and travelers’ vaccines, as well as the availability of vaccination programs for pregnant women for such vaccines and their development for pregnant women [5,6].

The decision on vaccination is usually based on the results of clinical trials. While conducting clinical trials on pregnant women for therapeutic drugs is challenging, the same is true for vaccines. There are few products for which the advisability of vaccination to pregnant women has been determined based on clinical trials.

In this study, we categorized the information concerning vaccination of pregnant women based on the package insert of all vaccines approved in Japan, the USA, and Europe. Moreover, we examined the information affecting vaccination decisions during pregnancy based on non-clinical studies, clinical trials, and information on pregnancy outcomes obtained during post-approval accidental maternal vaccination. The results revealed the approach of the regulatory authorities in decision making regarding vaccination during pregnancy. In addition, we listed the vaccines against SARS-CoV-2 as cases of vaccine development that took into account vaccination of pregnant women prior to approval. Furthermore, we compared the information from the studies on vaccination for SARS-CoV-2 during pregnancy against the vaccine development guidelines for pregnant women laid down by the World Health Organization (WHO) and European Medicines Agency (EMA). Based on our analyses, we summarized the points for making decisions regarding vaccination during pregnancy and hence, the information to be collected prior to vaccination. By referring to these findings, it is expected that clear decisions can be made regarding vaccination during pregnancy and vaccines can be developed promptly for pregnant women after conducting necessary and sufficient studies, such as non-clinical reproductive and developmental toxicity studies, and clinical trials including non-pregnant women of childbearing age, when vaccination during pregnancy is absolutely necessary.

## 2. Methods

### 2.1. Classification for Deciding on Vaccination during Pregnancy

We investigated the contents of the Japanese and American package inserts and summary of product characteristics in Europe as of 1 November 2021, for vaccines approved in Japan, the USA, and Europe (Table 1) from 1 January 2000 to 30 November 2021, to clarify what information is provided on vaccination during pregnancy. We classified the contents into the following eight categories: (a) the vaccine should be administered during pregnancy only when clearly needed, (b) the vaccine should be administered during pregnancy only if the potential benefits outweigh the potential risks, (c) Pregnant women are recommended to postpone or interrupt vaccination until completion of pregnancy, (d) as a precautionary measure, vaccination should be avoided during pregnancy, (e) the vaccine is contraindicated during pregnancy, (f) the vaccine is not intended for women of childbearing age, (g) only background information is provided, but the decision on vaccination during pregnancy is not included, and (h) missing information on administration of the vaccine to pregnant women.

In addition, we investigated the results of non-clinical reproductive and developmental toxicity studies based on Japanese review reports, review documents in the USA, and European public assessment report (EPAR) of each approved vaccine and compared to the abovementioned eight categories to explore the basis of decision-making on vaccination during pregnancy.

### 2.2. Vaccine Development Guidelines and SARS-CoV-2 Vaccine Response

The WHO, 2017, Guidelines on clinical evaluation of vaccines: regulatory expectations [7] and EMA, 2018, Guideline on Clinical Evaluation of Vaccines Draft [8] include points of consideration for vaccination during pregnancy. The content of these guidelines was compared.

As of 26 December 2021 the vaccines against the SARS-CoV-2 virus approved by the EMA, United States Food and Drug Administration, or Pharmaceuticals and Medical Devices Agency in Japan were: Comirnaty^®^ [9,10,11], Spikevax^®^ [12,13], Vaxzevria^®^ [14,15], and Janssen COVID-19 vaccine [16]. We investigated the non-clinical studies and clinical trials conducted for each vaccine and compared these against the WHO and EMA guidelines for pregnant women. We also investigated the post-approval studies or surveys which collected information on pregnancy outcomes obtained from maternal vaccination by accessing the Risk Management Plan in Japan, Pharmacovigilance Planning or Pharmacovigilance Activities in the USA, and Risk Management Plan in Europe, and discussed improvement measures for collecting information related to vaccination during pregnancy.

## 3. Results

### 3.1. Classification for Deciding on Vaccination during Pregnancy

The classification categories indicating the decision to vaccinate during pregnancy were different in Japan, the USA, and Europe. Europe used the largest number of classification categories, ranging from (a) to (g), described in the Methods section. The category (b), i.e., the vaccine should be administered during pregnancy only if the potential benefits outweigh the potential risks, was indicated most frequently, accounting for 26.0% (12 out of 46 vaccines). This was followed by category (a), i.e., the vaccine should be administered during pregnancy only when clearly needed, accounting for 23.9% (11 out of 46 vaccines). The USA followed five categories, (a) and (e)–(h), wherein category (g), i.e., only background information is provided, but the decision on vaccination during pregnancy is not included, was most common at 40.0% (30 out of 75 vaccines). Japan followed four categories, (a), (c), (e), and (h), and category (a) accounted for the majority at 63.0% (29 out of 46 vaccines).

**Figure 1:**
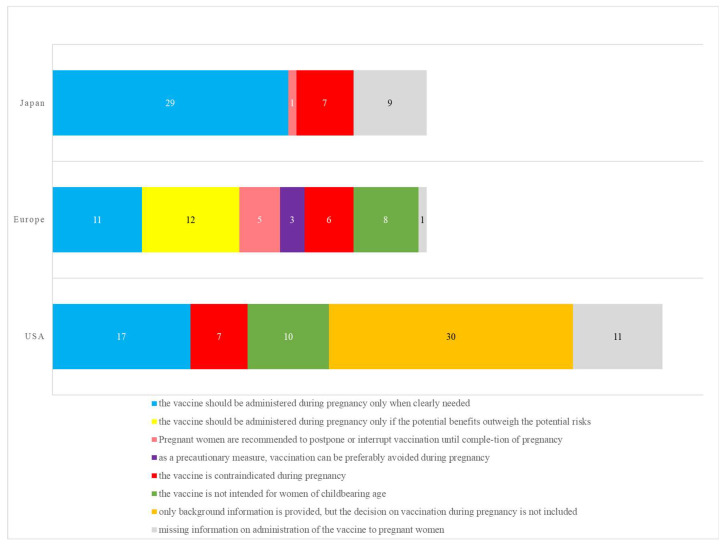
Classification categories influencing the decision of vaccination during pregnancy in each country or region. The number in the graph is the number of vaccines.

Based on the results of the non-clinical reproductive and developmental toxicity studies conducted on the vaccines approved in the USA, the only product showing an abnormality was the Japanese encephalitis vaccine, Ixiaro^®^ [17]. Ixiaro^®^ showed a statistical preponderance of incomplete ossification of the fetus in the studied groups, but a causal relationship between this abnormality and the vaccine was not clear; hence, the vaccine was considered available if required. In addition, the USA registry for maternal vaccination provided information on 16 out of 75 vaccines (21.3%) in the USA, but none of them showed any major abnormalities or were similar to the estimated background rate of congenital abnormalities (Figure 1). In Europe, out of 32 vaccines (69.6%), for which information was provided, only one vaccine, Ixiaro^®^, showed abnormalities, similar to those observed in the USA. In terms of the vaccination experience of pregnant women, 11 vaccines (23.9%) provided desirable results without any abnormalities.

The live attenuated vaccines, including measles, rubella, mumps, varicella, shingles, and adenovirus, are contraindicated in all countries or regions. In particular rubella [18], mumps [18], and varicella virus [19] are known to cause adverse effects on the fetus, including miscarriage, due to infection by the wild-type virus during pregnancy.

The yellow fever and rabies vaccines approved in Japan and the USA, the Ebola virus vaccine approved in Europe and the USA, and the anthrax and salmonella vaccines approved in the USA should be considered for vaccination only if clearly needed. Although the yellow fever vaccine is a live attenuated vaccine, it has been given to many pregnant women without any apparent adverse effects on the fetus [20].

The yellow fever vaccine in Japan is the same product as that in the USA. Based on the ACIP [21] and literature [22], it provides the information of the possibility of transplacental transmission of the vaccine-derived virus, however, the basis for the decision that it could be administered when necessary was not clear. We surmise that the decision may have been made by referring to the discussions and handling of the product in the USA, where it was approved earlier.

Regarding COVID-19 vaccine, cases of thromboembolic events have been reported following administration of Vaxzevria^®^ in several European countries. The EMA conclude that there was currently no evidence to suggest an association of thrombotic events following the use of Vaxzevria^®^, based on the review of clinical and non-clinical data [23]. Although these events occurred mainly in women younger than 55 years of age, these phenomena may reflect a bias in the vaccinated population. As a consequence, this review did not have a specific impact on the information provided for vaccination of pregnant women.

In the USA, for both the COVID-19 vaccine “PFIZER-BIONTECH” and COVID-19 vaccine “MODERNA” classified (g) only background information is provided, however the decision on vaccination during pregnancy is not included. No abnormalities were observed at reproductive and developmental toxicity studies for either vaccine.

There have been cases where vaccines of the same modality targeting the same disease were classified differently within the same country or region or among different countries or regions. In Europe, the intranasal live seasonal influenza vaccine [24] was contraindicated in pregnant women, whereas no adverse events were noted in the non-clinical reproductive and developmental toxicity studies and post-marketing data on the maternal vaccination registry. In contrast, the intranasal live vaccine for the highly virulent H5N1 influenza [25], with the same manufacturing process as the aforementioned seasonal influenza vaccine, was authorized for administration during pregnancy if the potential benefits outweighed the potential risks.

Although Ixiaro^®^ was approved both in Europe and the USA, it was approved for vaccination during pregnancy in the USA if the need for vaccination outweighed the risks [17], while in Europe, its administration was avoided as a precautionary measure [26]. However, in the USA, the results of a non-clinical reproductive and developmental toxicity study were resubmitted in 2017 [27], in which no fetal abnormalities were observed.

### 3.2. Vaccine Development Guidelines and SARS-CoV-2 Vaccine Response

#### 3.2.1. Description of the Guidelines

Both the WHO [7] and EMA [8] guidelines state that not all vaccines are suitable for administration to pregnant women. However, the WHO guidelines emphasize the importance of generating data for vaccination in pregnant women, whenever the target population for a vaccine includes women of childbearing age. For this purpose, the points to be considered are the modality of the vaccine (for example, the live attenuated vaccines), the possibility of avoiding exposure to an infectious agent, and the nature of the target infectious disease (for example, the greater risk of experiencing severe disease by pregnant women compared to non-pregnant women of the same age). On the other hand, both guidelines state that vaccination during pregnancy may have some specific aims, such as protecting the pregnant individual, protecting the fetus from intra-uterine infection, and protecting the infants as long as protective levels of maternal antibodies persist in the postnatal period. Additionally, the EMA guidelines state that clinical trials to select the dose regimens should enroll the women at a stage of pregnancy appropriate to each objective, for example: as early as possible in pregnancy to protect the mother and/or fetus and later in pregnancy to maximize the maternal antibody levels in the neonate.

Before conducting trials in pregnant women, both guidelines indicate that the vaccine should have been assessed via appropriate non-clinical studies, and the safety and immunogenicity data should be available from clinical trials conducted in non-pregnant women of childbearing age. Furthermore, they noted that if the immune correlate of protection (ICP) is established for the targeted infectious disease, the maternal vaccination regimen should maximize the antibody proportion in order for it to exceed the ICP in pregnant women or cord blood samples. In addition, the EMA guidelines state that the effect of the time interval between vaccination and delivery of maternal antibody levels in infants should be assessed, if the primary aim of the vaccination during pregnancy is to protect the infant. The WHO guidelines state the importance of the antibody decay curve in cases where the passive protection by the maternal antibody will be followed by active vaccination of the infants using the same antigen. This can potentially avoid the possibility of interference by the high levels of maternal antibody with the infant immune response. Finally, both guidelines emphasize the collection of pregnancy results (duration of gestation at the time of delivery, condition of the infant at birth, and presence of any congenital conditions), if the pregnant woman is vaccinated.

#### 3.2.2. Investigation of SARS-CoV-2 Vaccination during Pregnancy

Since there is a serious concern about the severity of COVID-19 in pregnant women [28], and studies are ongoing on the transfer of vaccine-derived maternal antibodies to newborns, we investigated the findings of the studies on maternal vaccination with Comirnaty^®^ [29,30], Spikevax^®^ [31], Vaxzevria^®^ [32] and the Janssen COVID-19 vaccine [16].

The non-clinical reproductive and developmental toxicity studies conducted for all the above vaccines revealed no abnormal findings. Hence, the vaccines were subjected to clinical trials involving non-pregnant women of childbearing age; however, by the time of approval, no clinical trial had been conducted in pregnant women. The information on vaccination during pregnancy was listed as important missing information in the risk management plans in the post-approval period. As an additional pharmacovigilance plan for all vaccines, registry enrollment or prospective cohort studies were planned to collect information on the safety and pregnancy outcomes for instances of vaccination during pregnancy (Table 2).

Clinical trials in pregnant women were planned for the Comirnaty^®^ and Janssen COVID-19 vaccines. A placebo-controlled, randomized, observer-blind study was conducted with Comirnaty^®^ [9,10] to compare the safety and antibody titer results in pregnant women with those in non-pregnant women of childbearing age. A single-arm open-label trial was conducted with the Janssen COVID-19 vaccine [16] to measure antibody titers in newborns using cord blood and serum.

## 4. Discussion

Most of the vaccines assessed in this study showed no abnormal side-effects in non-clinical reproductive and developmental toxicity studies. Furthermore, the post-marketing information revealed no abnormalities that exceeded background values in cases of vaccination during pregnancy. However, during pregnancy, some of the vaccines were contraindicated or avoided as a precautionary measure.

A few live vaccines, although attenuated, were contraindicated, because the wild-type viruses caused congenital abnormalities or severe illness in pregnant women. Non-clinical reproductive and developmental toxicity studies have not been conducted for such vaccines. Moreover, other live attenuated vaccines with wild-type pathogens against infectious diseases that have not been shown to have adverse effects in pregnant women were also contraindicated, because they were a combination of other live vaccines that were contraindicated for pregnant women. However, as an exception, the yellow fever vaccine was authorized for administration during pregnancy when the need for vaccination exceeded the assumed risk as well as factors, such as the difficulty of planned vaccination due to challenges in accessing endemic areas and the severity of the disease affecting the prognosis of the pregnant women and their fetuses. Therefore, vaccination is authorized when the necessity outweighs the assumed risk.

Influenza is not considered teratogenic, and no adverse effects have been demonstrated in non-clinical reproductive and developmental toxicity studies of the influenza vaccines. The live intranasal vaccine for seasonal influenza, approved by the EMA in 2013, was not recommended for vaccination during pregnancy, as live vaccines are generally not indicated for pregnant women. In contrast, the vaccine for the highly pathogenic H5N1 influenza was approved in 2016 and authorized for administration during pregnancy. This is mainly because the USA Vaccine Adverse Event Reporting System or voluntary reports for seasonal vaccines could not identify any congenital anomalies resulting from the vaccine. Although the H5N1 influenza is associated with death and other serious illnesses among pregnant women [33], the need for vaccination in the event of a pandemic is high due to its high pathogenicity.

In developing a vaccine, it is necessary to evaluate the characteristics, occurrence, and prevalence of the target disease in the target country or region and conduct non-clinical reproductive and developmental toxicity studies in order to assess the vaccination needs during pregnancy. It is also necessary to extract relevant information from the results of the vaccination during pregnancy using approved vaccines for the same target disease and vaccines with similar modalities. If vaccination during pregnancy is essential, in addition to a non-clinical reproductive and developmental toxicity study, a clinical trial on pregnant women must be conducted with reference to the results of clinical trials in healthy non-pregnant women of childbearing age. Despite the WHO and EMA guidelines, most vaccines have not provided the results of clinical trials for pregnant women in the package inserts. The implementation of clinical trials for pregnant women has been delayed for vaccines, and therapeutic drugs [34]. In the case of vaccines, clinical trials for pregnant women have not been conducted, even when there are concerns that pregnant women have more severity of the disease than the non-pregnant population, as in the case of H1N1 influenza [32]. However, clinical trials for some COVID-19 vaccines are being conducted after their approval.

In many cases, the surveys or clinical trials for pregnant women were not conducted before the approval of a new active-ingredient vaccine, and information collection and clinical trials on the vaccination of pregnant women were conducted as a part of the post-marketing risk management plan.

In particular, for emerging infectious diseases, such as H1N1 influenza in 2009 and COVID-19 in 2019, it is difficult to vaccinate systematically in advance of pregnancy, as is conventionally done for the existing infectious diseases. Furthermore, the need for vaccination during pregnancy was recognized, because severe cases of both diseases were observed in pregnant women. For COVID-19, immunization of newborns via the placenta was expected, whereas for the H1N1 influenza vaccine, there was a certain amount of experience with vaccination during pregnancy with conventional seasonal influenza vaccines, which are of the same modality as the H1N1 influenza but different strains. The SARS-CoV-2 vaccine is based on new modalities such as mRNA or DNA vaccines; hence, the need for evaluating the effects of such vaccines during pregnancy has been recognized.

The WHO [7] and EMA [8] guidelines state that information on pregnancy outcomes should be collected for vaccination during pregnancy programs along with recommendations for evaluation by clinical trials. In line with the WHO and EMA guidelines, non-clinical reproductive and developmental toxicity studies and clinical trials involving non-pregnant women of childbearing age were conducted during the development of the SARS-CoV-2 vaccine [29,30,31,32]. In addition, information on cases of accidental vaccination during pregnancy in pre-approval clinical trials, including pregnancy outcomes, has been obtained from existing registries and other resources on vaccine administration to pregnant women. While the WHO [7] and EMA [8] guidelines recommend clinical trials in pregnant women, such trials and measurement of antibody titers in newborns using cord blood at delivery were performed only for a few vaccines. The information on the safety and pregnancy outcomes at the time of vaccination was more actively collected through observational studies such as registries and cohort studies. Expanding the target age range for vaccination against COVID-19 to include children, but not neonates or infants, is being considered. It is preferable to estimate the antibody titer in newborns using cord blood at delivery, as this technique is noninvasive.

Although clinical trials for pregnant women can provide detailed information, such as antibody titer from the cord blood of newborns, it has rarely been implemented, and even if it were, the number of study participants is usually limited. Furthermore, the number of cases required to evaluate the time of vaccination in terms of the number of weeks into pregnancy and the associated the safety and frequency of pregnancy outcomes is often insufficient. The use of cohort studies is considered to be effective based on a comparison of previously obtained data.

For vaccination during pregnancy and the collection of information in the post-marketing period, it is desirable to conduct a survey with international coordination, especially in view of the limited information on post-vaccination outcomes and increasing globalization of vaccine development. Most package inserts of the USA and Europe provide information on the results of non-clinical reproductive and developmental toxicity studies and registry data on vaccination during pregnancy. They also compare these data with the background of non-vaccinated individuals with birth defects. These data are required when the need for vaccination during pregnancy arises, or when an accidental vaccination occurs, such as the detection of pregnancy after vaccination. In Japan, we recommend collecting information on vaccination during pregnancy and providing such information in the package insert or in an interview form as a supplementary document.

## 5. Conclusions

In a planned immunization program, it is desirable to vaccinate before pregnancy as much as possible. However, for vaccinations that cannot be avoided during pregnancy, such as vaccines for emerging and reemerging infectious diseases and travelers’ vaccines, registry surveys and other observational studies have been conducted to obtain information on the safety of the pregnant women themselves and the newborn. It is also necessary to obtain the results of non-clinical reproductive and developmental toxicity studies and clinical trials in women of childbearing age before the approval of the vaccine. Considering the globalization of vaccine development, international coordination for non-clinical studies and clinical trials is beneficial.

## Figures and Tables

**Table 1:** List of vaccines examined in this study.

Product Name	Trade Name (Japan)	Trade Name (USA)	Trade Name (Europe)
Pneumococcal 10-valent conjugate vaccine	—	—	Synflorix^®^ (f)
Pneumococcal 13-valent conjugate vaccine	Prevenar 13^®^ (a)	Prevenar 13^®^ (g)	Prevenar 13^®^ (e)
Pneumococcal 15-valent conjugate vaccine	—	Vaxneuvance^®^ (g)	—
Pneumococcal 20-valent conjugate vaccine	—	Prevenar 20^®^ (g)	—
Pnemococcal vaccine, polyvalent	PneumovaxNP^®^ (a)	Pneumovax 23^®^ (g)	—
Hepatitis A vaccine	Aimmugen^®^ (a)	Havrix^®^ (g)	—
		Vaqta^®^ (g)	
BCG vaccine	Freeze-dried glutamate BCG vaccine for percutaneous use (a)	BCG vaccine (e)	—
		TICE BCG^®^ (e)	—
Hepatitis B vaccine	Bimmugen^®^ (a)	Engerix-B^®^ (a)	HBVaxPro^®^ (b)
	Heptavax-II^®^ (a)	Recpmbivax HB^®^ (g)	Fendrix^®^ (b)
		Heplisav B^®^ (g)	Heplisav B^®^ (b)
		Hepatitis B surface antigen (h)	—
Hepatitis A and Hepatitis B vaccine	—	Twinrix^®^ (g)	Ambirix^®^ (a)
			Twinrix Adult^®^ (d)
			Twinrix Paediatric^®^ (d)
Diphtheria, tetanus toxoids, and acellular pertussis adsorbed and inactivated poliovirus vaccine	Quattrovac^®^ (h)	Kinrix^®^ (h)	—
	Tetrabik^®^ (h)	Quadracel^®^ (h)	
	SquareKids^®^ (h)		
Product Name	Trade Name (Japan)	Trade Name (USA)	Trade Name (Europe)
Diphtheria, tetanus toxoids, and acellular pertussis adsorbed, inactivated poliovirus and Haemophilus b conjugate (Tetanus toxoid conjugate) vaccine	—	Pentacel^®^ (h)	—
Diphtheria, tetanus toxoids, and acellular pertussis adsorbed, inactivated poliovirus, Haemophilus b conjugate (Meningococcal protein conjugate), and Hepatitis B (recombinant) vaccine	—	Vaxelis^®^ (f)	Vaxelis^®^ (f)
			Hexacima^®^ (f)
			Infanrix Hexa^®^ (f)
			Hexyon^®^ (f)
Diphtheria, tetanus toxoids, and acellular pertussis adsorbed vaccine	Tribik^®^ (a)	Boostrix^®^ (g)	—
		Infanrix^®^ (h)	
		Adacel Tdap^®^ (g)	
		Daptacel^®^ (f)	
		Diphteria and tetanus toxoids adsorbed (e)	
Diphtheria, tetanus toxoids, and acellular pertussis adsorbed vaccine, Hepatitis B (recombinant) and inactivated poliovirus combined vaccine	—	Pediarix^®^ (h)	—
Diphtheria and tetanus toxoids adsorbed vaccine	DTBik^®^ (h)	Tdvax^®^ (a)	—
	Diphtheria and tetanus toxoids “TAKEDA” (h)	Tetanus and diphtheria toxoids adsorbed injection (a)	
Human papillomavirus vaccine	Gardasil^®^ (a)	Gardasil^®^ (a)	—Gardasil^®^ (c)
Product Name	Trade Name (Japan)	Trade Name (USA)	Trade Name (Europe)
Human papillomavirus vaccine	Silgard9^®^ (a)	Gardasil9^®^ (g)	Gardasil 9^®^ (c)
	Cervarix^®^ (c)		Cervarix^®^ (c)
Adenovirus vaccine	—	Live kit adenovirus Type 4 and Type 7 vaccine (e)	—
Yellow fever vaccine	YF-Vax^®^ (a)	YF-Vax^®^ (a)	—
		Stamaril ^®^(a)	
Mumps vaccine	Freeze-dried live attenuated mumps vaccine “DAIICHI SANKYO” (e)	—	―
Haemophilus b conjugate vaccine	ActHIB^®^ (h)	ActHIB^®^ (f)	—
		PedvaxHIB^®^ (f)	
		Hiberix^®^ (f)	
		Haemophilus influenza Type B conjugate HIB (f)	
Influenza vaccine	Influenza HA vaccine “BIKEN” (a)	Fluad Quadrivalent^®^ (f)	Supemtek^®^ (b)
	Flubik HA^®^ (a)	Fluarix Quadrivalent^®^ (g)	Aflunov^®^ (b)
	Influenza HA vaccine “KMB” (a)	Flulaval Quadrivalent^®^ (g)	Fluad Tetra^®^ (e)
	Influenza HA vaccine “SEIKEN” (a)	Flumist Quadrivalent^®^ (a)	Fluenz Tetra^®^ (f)
	Influenza HA vaccine “DAIICHI SANKYO” (a)	Influenza A-H1N1 monovalent vaccine (a)	
		Afluria Quadrivalent^®^ (g)	
		Flubik Quadrivalent Northern Hemisphere^®^ (g)	
		Flucelvax Quadrivalent^®^ (g)	
		Fluvirin^®^ (h)	
		Fluzone High-Dose Quadrivalent Northern Hemisphere^®^ (f)	
Product Name	Trade Name (Japan)	Trade Name (USA)	Trade Name (Europe)
Influenza vaccine (continue)		Fluzone Quadrivalent^®^ (g)	
		Influenzinum^®^ (h)	
		Medical provider single use EZ flu shot^®^ (h)	
			Pandemic influenza vaccine H5N1 “ASTRAZENECA” (b)
			Foclivia^®^ (b)
			Adjupanrix^®^
			Pandemic influenza vaccine H5N1 “BAXTER AG” (a)
Rabies vaccine	Rabipur^®^ (a)	Rabavert^®^ (a)	—
	Inactivated tissue culture rabies vaccine (a)	Imovax Rabies^®^ (a)	
Ebola Zaire vaccine	—	Ervebo^®^ (a)	Ervebo^®^ (a)
			Mvabea^®^ (a)
			Zabdeno^®^ (a)
Cholera vaccine	—	Vaxchora^®^ (a)	Vaxchora^®^ (b)
			Dukoral^®^ (g)
Typhoid vaccine	—	Typhim Vi^®^ (a)	—
		Vivotif ^®^(a)	
COVID-19 vaccine	Comirnaty^®^ (a)	COVID-19 vaccine “PFIZER-BIONTECH” (g)	Comirnaty^®^ (b)
	COVID-19 vaccine “MODERNA” (a)	COVID-19 vaccine “MODERNA” (g)	COVID-19 vaccine “JANSSEN” (b)
		COVID-19 vaccine “JANSSEN” (g)	Spikevax^®^ (previously COVID-19 vaccine “MODERNA”) (b)
		Antigen component (h)	Vaxzevria^®^ (b)
Product Name	Trade Name (Japan)	Trade Name (USA)	Trade Name (Europe)
Meningococcal conjugate vaccine	Menactra^®^ (a)	Menactra^®^ (g)	Nimenrix^®^ (a)
		Menveo^®^ (g)	Menveo^®^ (a)
		Bexsero^®^ (g)	Bexero^®^ (a)
		MenQuadfi^®^ (g)	MenQuadifi^®^ (a)
		Trumenba^®^ (g)	Trumenba^®^ (a)
Zoster vaccine	Shingrix^®^ (a)	Shingrix^®^ (g)	Shingrix^®^ (d)
		Zostavax^®^ (e)	Zostavax^®^ (e)
Tuberculin skin test	Freeze-dried tuberculin purified protein derivative (h)	—	—
Tick-borne encephalitis vaccine	—	Ticovac^®^ (g)	—
Anthrax vaccine	—	Biothrax^®^ (a)	—
Dengue vaccine	—	Dengvaxia^®^ (g)	Dengvaxia^®^ (e)
Smallpox vaccine	—	ACAM2000^®^ (a)	Imvanex^®^ (d)
Japanese encephalitis virus vaccine	Encevac^®^ (a)	Ixiaro^®^ (a)	Ixiaro^®^ (d)
	Jebik V^®^ (a)		
Measles, mumps, rubella, and varicella virus live vaccine	—	Proquad^®^ (e)	—
Tetanus and diphtheria toxoids adsorbed vaccine	Adsorbed tetanus toxoid “SEIKEN” (a)	Tenivac^®^ (g)	—
	Adsorbed tetanus toxoid “KAKEDA” (a)		
	Tetanus toxoid “BIKEN F” (a)		
Rubella vaccine	Dried live attenuated rubella vaccine “TAKEDA” (e)	—	—
Measles, mumps, and rubella virus vaccine	—	M-M-R II^®^ (e)	—
Poliovirus vaccine	Imovax Polio^®^ (h)	IPOL^®^ (a)	
Product Name	Trade Name (Japan)	Trade Name (USA)	Trade Name (Europe)
Measles and rubella vaccine	Mearubik (e)	—	MM-R VaxPro^®^ (e)
	Freeze-dried live attenuated measles and rubella combined vaccine “DAIICHI SANKYO” (e)		
	Freeze-dried live attenuated measles and rubella combined vaccine “TAKEDA” (e)		
	Freeze-dried live attenuated measles and rubella combined vaccine “HOKKEN” (e)		
Measles vaccine	Dried live attenuated measles vaccine (e)	―	
Measles, mumps, rubella, and varicella virus vaccine	—	―	ProQuad^®^ (e)
Rotavirus vaccine	Rotarix^®^ (h)	Rotarix^®^ (h)	Rotarix^®^ (f)
	RotaTeq^®^ (h)	RotaTeq^®^ (f)	RotaTeq^®^ (f)

(a) The vaccine should be administered during pregnancy only when clearly needed, (b) the vaccine should be administered during pregnancy only if the potential benefits outweigh the potential risks, (c) pregnant women are recommended to postpone or interrupt vaccination until completion of pregnancy, (d) as a precautionary measure, vaccination should be avoided during pregnancy, (e) the vaccine is contraindicated during pregnancy, (f) the vaccine is not intended for women of childbearing age, (g) only background information is provided, but the decision on vaccination during pregnancy is not included, and (h) missing information on administration of the vaccine to pregnant women. ^®^ is the registered trademark.

**Table 2:** Post-approval surveillance of pregnant women receiving the SARS-CoV-2 vaccine.

Comirnaty^®^	Spikevax^®^	Vaxzevria^®^	Janssen COVID-19 Vaccine
Clinical trial for pregnant women (USA)	Real-world effectiveness study (USA)		Clinical trial for pregnant women (Europe)
Observational studies using existing data sources (USA, Europe)	Observational studies using existing data sources (Europe)	Observational studies using existing data sources (Europe)	Observational studies using existing data sources (Europe)
Registry investigation (USA, Europe)	Registry investigation (USA, Europe)	Registry Investigation (Europe)	Registry investigation (Europe)
Cohort study (USA)			Observational study (USA)
Use-results survey (Japan)	Use-results survey (Japan)	Use-results survey (Japan)	

The country or region of implementation is indicated in parenthesis. ^®^ is the registered trademark.

## Data Availability

Not applicable.
